# Supplementary material for: Aberrant oscillatory activity in neurofibromatosis type 1: an EEG study of resting state and working memory
Source: J Neurodev Disord. 2023 Aug 22;15:27. doi: 10.1186/s11689-023-09492-y (PMC10463416; doi:10.1186/s11689-023-09492-y)
Supplement: Supplementary file 1 — Additional file 1: Sub-sample demographic and behavioural information. Table 1. Descriptive and inferential statistics for age, sex, Vineland ABC scores, and auditory n-back performance. Table 2. Descriptive and inferential statistics for EEG visual n-back task performance (hits % – false alarms %). [file 11689_2023_9492_MOESM1_ESM.pdf]

Additional file 1

Sub-sample demographic and behavioural information

Table 1. Descriptive and inferential statistics for age, sex, Vineland ABC scores, and auditory n-back performance.

	Data	Descriptives CON	Descriptives NF1	*t*-test or Chi-square test
**Demographics**				
Age (M±SD (range))	Resting state	13.34±1.61 (11.33-16.92)	13.09±1.69 (11.25-16.58)	$t_{(28)} = 0.412$, p=.683
	Task-related (unadjusted)	13.34±1.61 (11.33-16.92)	13.05±1.72 (11.25-16.58)	$t_{(29)} = 0.483$, p=.633
	Task-specific (adjusted)	13.34±1.61 (11.33-16.92)	13.12±1.76 (11.25-16.58)	$t_{(27)} = 0.346$, p=.732
Sex (male/female)	Resting state	9/7	8/6	$\chi2 = 0.002$, p=.961
	Task-related (unadjusted)	9/7	8/7	$\chi2 = 0.027$, p=.870
	Task-specific (adjusted)	9/7	7/6	$\chi2 = 0.017$, p=.897
**Behavioural**				
Vineland ABC[1]	Resting state	103.56±15.35	81.85±14.31	$t_{(27)} = 3.904$, p=.001
	Task-related (unadjusted)	103.56±15.35	85.00±15.36	$t_{(28)} = 3.303$, p=.003
	Task-specific (adjusted)	103.56±15.35	84.00±12.56	$t_{(26)} = 3.598$, p=.001
Auditory n-back (mean n-back)	Resting state	2.64±0.38	1.98±0.36	$t_{(28)} = 4.872$, p=<.001
	Task-related (unadjusted)	2.64±0.38	1.91±0.37	$t_{(29)} = 5.461$, p=<.001
	Task-specific (adjusted)	2.64±0.38	1.96±0.37	$t_{(27)} = 4.884$, p=<.001

Abbreviations: M: mean, SD: standard deviation.

[1] One participant in the NF1 group did not have an ABC score.

Table 2. Descriptive and inferential statistics for EEG visual n-back task performance (hits % – false alarms %).

Data	1-back		2-back		ANOVA								
	CON	NF1	CON	NF1	Group			Load			Group x load		
	M±SD (%)	M±SD (%)	M±SD (%)	M±SD (%)	*F*	*p*	η_p^2	*F*	*p*	η_p^2	*F*	*p*	η_p^2
Resting-state	83.81±17.50	83.56±16.18	64.69±22.21	61.19±21.46	0.055	.816	.002	36.097	<.001	.563	0.057	.812	.002
Task-related (unadjusted)	83.81±17.50	84.00±16.65	64.69±22.21	61.20±22.22	0.068	.796	.002	41.167	<.001	.587	0.316	.578	.011
Task-specific (adjusted)	83.81±17.50	83.54±17.92	64.69±22.21	62.46±23.37	0.034	.855	.001	34.136	<.001	.558	0.080	.779	.003

Abbreviations: M: mean, SD: standard deviation.
