# Supplementary material for: Aberrant oscillatory activity in neurofibromatosis type 1: an EEG study of resting state and working memory
Source: J Neurodev Disord. 2023 Aug 22;15:27. doi: 10.1186/s11689-023-09492-y (PMC10463416; doi:10.1186/s11689-023-09492-y)
Supplement: Supplementary file 2 — Additional file 2: Statistical analysis of task-related unadjusted power and theta coherence. Table 1. Power (unadjusted): descriptive and inferential statistics. Table 2. Theta phase coherence (unadjusted): descriptive and inferential statistics. [file 11689_2023_9492_MOESM2_ESM.pdf]

Additional file 2

Statistical analysis of task-related *unadjusted* power and theta coherence

Table 1. Power *(unadjusted)*: descriptive and inferential statistics.

	1-back		2-back		ANOVA								
	CON	NF1	CON	NF1	Group			Load			Group x load		
	M±SD	M±SD	M±SD	M±SD	$F_{(1,29)}$	p	η_p^2	$F_{(1,29)}$	p	η_p^2	$F_{(1,29)}$	p	η_p^2
Delta	0.51±0.39	0.77±0.53	0.59±0.48	0.82±0.59	2.051	.163	.066	2.236	.146	.072	0.122	.729	.004
Theta	-0.63±0.47	-0.20±0.64	-0.59±0.51	-0.20±0.64	4.092	.052	.124	0.374	.545	.013	0.465	.501	.016
Alpha	-0.93±0.74	-0.48±0.73	-0.87±0.79	-0.52±0.73	2.296	.141	.073	0.053	.819	.002	1.675	.206	.055
Beta	-2.32±0.45	-2.25±0.50	-2.25±0.50	-2.24±0.48	0.058	.812	.002	4.136	.051	.125	1.836	.186	.060
Low gamma	-3.84±0.41	-3.64±0.65	-3.76±0.51	-3.61±0.56	0.848	.365	.028	2.005	.167	.065	0.358	.554	.012
High gamma	-4.98±0.53	-4.67±0.84	-4.86±0.66	-4.67±0.68	1.117	.299	.037	0.814	.374	.027	0.856	.363	.029
Mid-frontal theta (Fz/F1/F2)	-0.42±0.59	-0.06±0.57	-0.35±0.57	-0.02±0.67	2.850	.102	.089	0.559	.461	.019	0.034	.855	.001

Abbreviations: M: mean, SD: standard deviation. Power is averaged over all EEG electrodes unless otherwise specified. Degrees of freedom $_{(1,28)}$.

Table 2. Theta phase coherence *(unadjusted)*: descriptive and inferential statistics.

Descriptive statistics				
**Region**	**Load**	**Group**	**M±SD**	
Mid-frontal – left-parietal (ML)	1-back	CON	0.109±0.028	
		NF1	0.133±0.049	
	2-back	CON	0.120±0.030	
		NF1	0.140±0.048	
Mid-frontal – mid-parietal (MM)	1-back	CON	0.140±0.037	
		NF1	0.150±0.047	
	2-back	CON	0.140±0.034	
		NF1	0.144±0.028	
Mid-frontal – right-parietal (MR)	1-back	CON	0.103±0.017	
		NF1	0.135±0.035	
	2-back	CON	0.113±0.022	
		NF1	0.149±0.044	
**ANOVA**		$F_{(1,29)/(2,58)}$	p	η_p^2
Group		4.852	.036*	.143
Region		5.736	.005**	.165
Load		2.865	.101	.090
Group x region		2.309	.108	.074
Group x load		0.114	.739	.004
Region x load		4.700	.013*	.139
Group x region x load		0.473	.625	.016

*Abbreviations: M: mean, SD: standard deviation. ***p<.001, **p<.01, *p<.05. Degrees of freedom:* $_{(1,29),\ (2,58)}$.
