# Supplementary material for: Aberrant oscillatory activity in neurofibromatosis type 1: an EEG study of resting state and working memory
Source: J Neurodev Disord. 2023 Aug 22;15:27. doi: 10.1186/s11689-023-09492-y (PMC10463416; doi:10.1186/s11689-023-09492-y)
Supplement: Supplementary file 3 — Additional file 3: Resting state analyses for mid-frontal theta power and theta phase coherence. Table 1. Resting state (eyes open) mid-frontal theta power: descriptive and inferential statistics. Table 2. Resting state (eyes open) theta phase coherence: descriptive and inferential statistics. [file 11689_2023_9492_MOESM3_ESM.pdf]

Additional file 3

Resting state analyses for mid-frontal theta power and theta phase coherence

The NF1 group showed significantly higher mid-frontal theta power **(Table 1)** and frontoparietal theta phase coherence **(Table 2)** during resting state (eyes open) relative to controls.

Table 1. Resting state (eyes open) mid-frontal theta power: descriptive and inferential statistics.

	**Descriptives**	***t*-test**		
**Group**	**M±SD**	$t_{(28)}$	***p***	**d**
CON	-0.45±0.40	2.619	.014*	0.93
NF1	0.02±0.58			

*Abbreviations: M: mean, SD: standard deviation. *p<.05. Degrees of freedom: (28).*

Table 2. Resting state (eyes open) theta phase coherence: descriptive and inferential statistics.

**Descriptive statistics**			
**Region**	**Group**	**M±SD**	
Mid-frontal – left-parietal (ML)	CON	0.122±0.021	
	NF1	0.159±0.062	
Mid-frontal – mid-parietal (MM)	CON	0.143±0.040	
	NF1	0.153±0.038	
Mid-frontal – right-parietal (MR)	CON	0.125±0.032	
	NF1	0.155±0.044	
**ANOVA**	$F_{(1,28)}$	***p***	η_p^2
Group[1]	4.329	.047*	.134
Region	0.725	.489	.025
Group x region	1.591	.213	.054

*Abbreviations: M: mean, SD: standard deviation. *p<.05. Degrees of freedom: (1,28).*

[1] One extreme outlier was identified in the CON group (ML) from inspection of a Box and Whisker plot. After removing this outlier, the findings stayed the same (i.e., a significant main effect of group, but no significant main effect of region or group x region interaction).
