# Supplementary material for: Aberrant oscillatory activity in neurofibromatosis type 1: an EEG study of resting state and working memory
Source: J Neurodev Disord. 2023 Aug 22;15:27. doi: 10.1186/s11689-023-09492-y (PMC10463416; doi:10.1186/s11689-023-09492-y)
Supplement: Supplementary file 4 — Additional file 4: Scatterplots. Fig. 1. Scatterplots between EEG measures and age. Fig. 2. Scatterplots between EEG measures and Vineland ABC scores. Fig. 3. Scatterplots between EEG measures and auditory n-back performance (working memory). [file 11689_2023_9492_MOESM4_ESM.pdf]

Additional file 4

Scatterplots

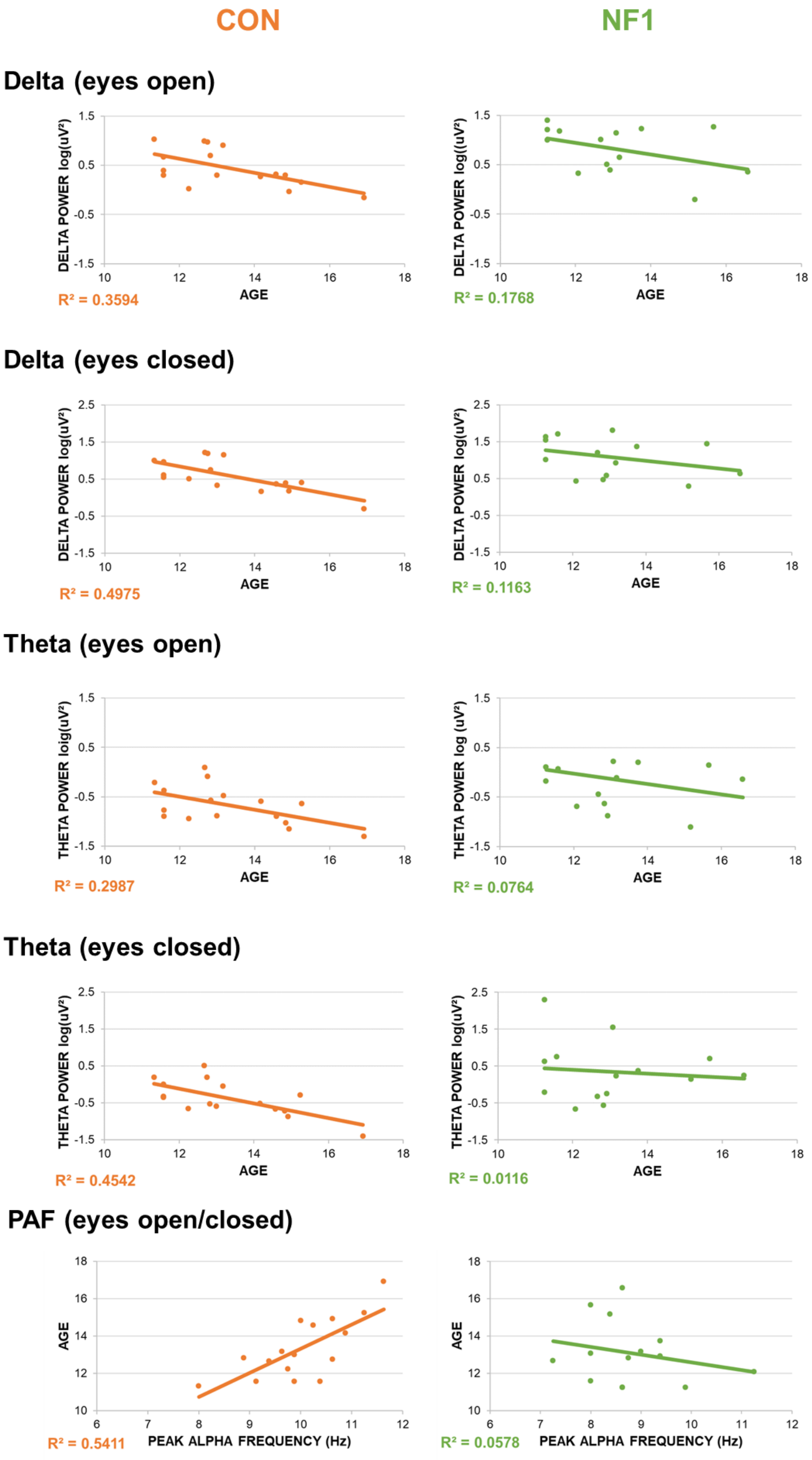

Fig. 1. Scatterplots between EEG measures and age.

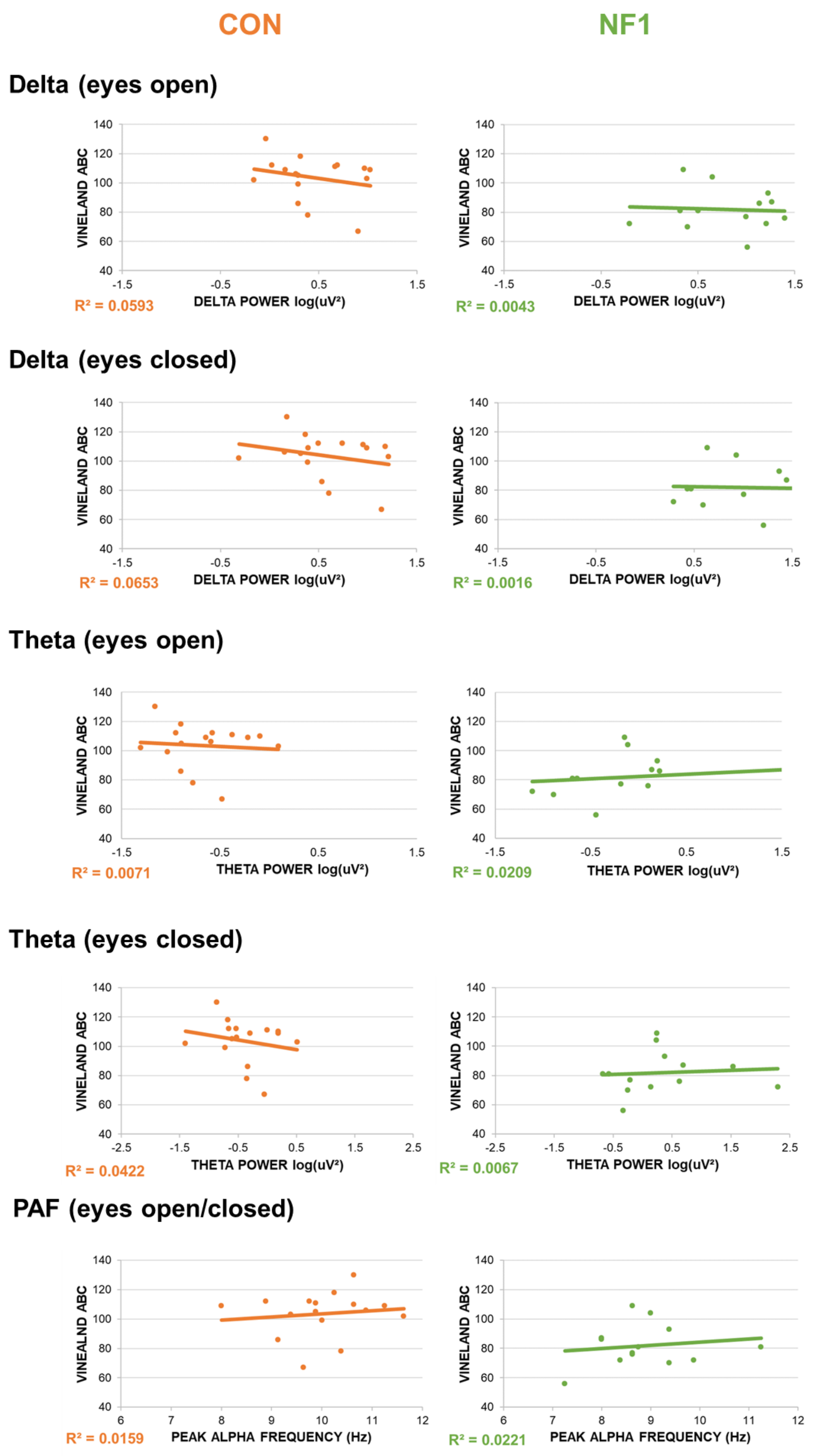

Fig. 2. Scatterplots between EEG measures and Vineland ABC scores (IQ).

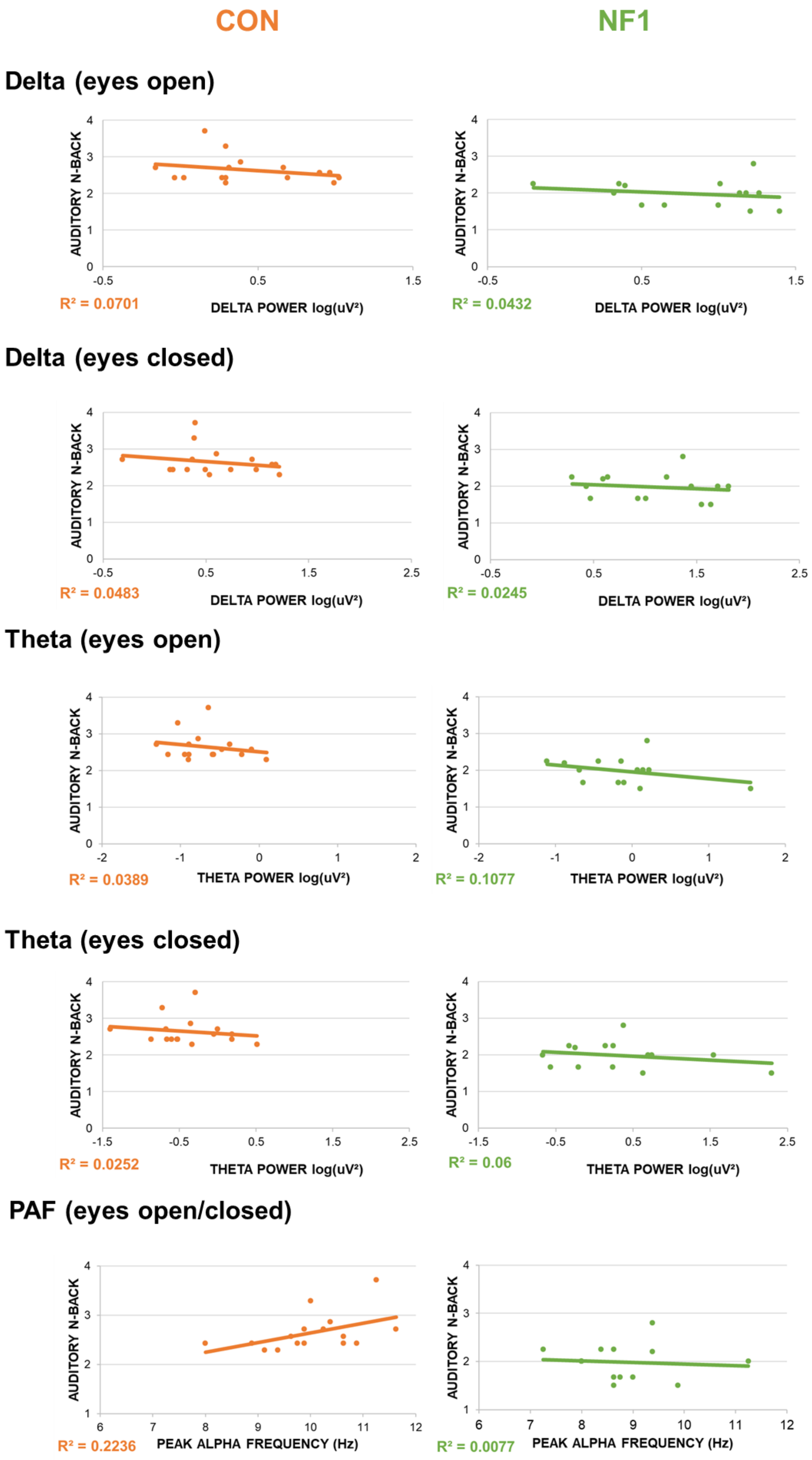

Fig. 3. Scatterplots between EEG measures and auditory n-back performance (working memory).
